# The hydrodynamics of jet propulsion swimming in hatchling and juvenile European common cuttlefish, *Sepia officinalis*

**DOI:** 10.1242/jeb.246225

**Published:** 2023-09-28

**Authors:** Nicholas W. Gladman, Graham N. Askew

**Affiliations:** School of Biomedical Sciences, Faculty of Biological Sciences, University of Leeds, Leeds LS2 9JT, UK

**Keywords:** Whole-cycle propulsive efficiency, Ontogeny, Cephalopod, Vortex rings

## Abstract

Cuttlefish swim using jet propulsion, taking a small volume of fluid into the mantle cavity before it is expelled through the siphon to generate thrust. Jet propulsion swimming has been shown to be more metabolically expensive than undulatory swimming, which has been suggested to be due to the lower efficiency of jet propulsion. The whole-cycle propulsive efficiency of cephalopod molluscs ranges from 38 to 76%, indicating that in some instances jet propulsion can be relatively efficient. Here, we determined the hydrodynamics of hatchling and juvenile cuttlefish during jet propulsion swimming to understand the characteristics of their jets, and whether their whole-cycle propulsive efficiency changes during development. Cuttlefish were found to utilise two jet types: isolated jet vortices (termed jet mode I) and elongated jets (leading edge vortex ring followed by a trailing jet; termed jet mode II). The use of these jet modes differed between the age classes, with newly hatched animals nearly exclusively utilising mode I jets, while juveniles showed no strong preferences. Whole-cycle propulsive efficiency was found to be high, ranging from 72 to 80%, and did not differ between age classes. During development, Strouhal number decreased as Reynolds number increased, which is consistent with animals adjusting their jetting behaviour in order to maximise whole-cycle propulsive efficiency and locomotor performance. Although jet propulsion swimming can have a relatively high energetic cost, in cuttlefish and nautilus, both neutrally buoyant species, the whole-cycle propulsive efficiency is actually relatively high.

## Teaser

**Summary:** Cuttlefish jet propulsion swimming utilises one of two jet modes to swim relatively efficiently; their jetting behaviour is optimised for maximal locomotor performance and efficiency throughout development.

## INTRODUCTION

Cuttlefish (Sepiidae) utilise a dual-mode locomotory system that involves both jet propulsion swimming, powered by the muscles of the mantle cavity, and undulatory swimming, powered by the undulations of the fins on the periphery of the mantle. These two locomotive modes are utilised both independently and simultaneously, giving cuttlefish flexibility in their swimming speed and manoeuvrability (Helmer et al., 2017; Jastrebsky et al., 2016). At high speeds, such as during escape responses, the locomotion of these animals is driven solely by jet propulsion (Staudinger et al., 2013), whereas at slow speeds, swimming by fin undulations is utilised. This switch to jet propulsion with increasing speed may incur an increase in metabolic energy expenditure, as the metabolic cost of transport (COT_met_) has been estimated to be up to 3.5 to 5 times greater in a jet-propelled squid compared with undulatory swimming fishes (Wells and O'Dor, 1991; Bartol et al., 2001). The underlying reason for the increased COT_met_ in jet propulsion compared with undulatory swimming is thought to be due to the difference in efficiency (i.e. the ratio of useful power output to the mechanical power input; Alexander, 2002). During jet propulsion swimming, relatively small volumes of fluid must be accelerated to much higher velocities to generate the same thrust as undulatory swimmers (Krieg and Mohseni, 2015; O'Dor and Webber, 1991; Weymouth and Triantafyllou, 2013). This results in a lower efficiency, requiring more metabolic energy to be expended to generate thrust. Gleiss et al. (2017) further noted a relationship between buoyancy and drag, where neutrally buoyant animals experience decreased drag and achieve greater efficiencies and lower COT_met_, particularly at lower swimming speeds. However, despite perceived inefficiencies, jet propulsion remains a key element in cephalopod locomotion. The efficiency of swimming has been quantified in several ways. For example, propulsive efficiency has been calculated as the ratio of mechanical power output to the mechanical power input (i.e. the sum of the mechanical power output and the rate at which kinetic energy is lost in the wake; Anderson and DeMont, 2000). Propulsive efficiency in cephalopod molluscs ranges from 70 to 93% in squid (Anderson and Grosenbaugh, 2005; Bartol et al., 2008, 2009a,b). However, this approach assumes an ideal and steady flow, which is inappropriate for cephalopod jet propulsion swimming in which flow is unsteady as the water is accelerated as it enters the mantle cavity and again as it is expelled (Alexander, 2002; Anderson and DeMont, 2000). Whole-cycle propulsive efficiency incorporates these unsteady effects and is defined as the ratio of mechanical power output to the sum of the mechanical power output and the rates at which kinetic energy is lost in the wake and during mantle cavity refilling (Alexander, 2002; Anderson and DeMont, 2000). Whole-cycle propulsive efficiency in cephalopod molluscs is lower (owing to the additional energy input during refilling) and ranges from 30 to 76% in nautilus (*Nautilus pompilius*; Neil and Askew, 2018) and 38 to 49% in squid (Anderson and Grosenbaugh, 2005 recalculated from Anderson and DeMont, 2000). The efficiency in cephalopod molluscs may depend, in part, on the nature of the jet structure itself. Two discrete types of jet can be produced by cephalopods, such as the brief squid (*Lolliguncula brevis*) and nautilus (*N. pompilius*), where single isolated jet vortices and elongated jets (leading edge vortex ring followed by a trailing jet) have been found (Bartol et al., 2008), with single isolated jet vortices associated with increased propulsive efficiency compared with the more elongate structures.

Jet propulsion swimming of European common cuttlefish (*Sepia officinalis*) is a key mode of the locomotion of these animals, of particular importance during escape responses. This propulsive system is fully developed when animals emerge from eggs and continues to be used throughout ontogeny into the adult stage. During ontogeny, the relative importance of the inertial and viscous forces experienced changes as the animal increases in size; the ratio of these forces is the Reynolds number (*Re*; Muller et al., 2008; Ngo and McHenry, 2014). The *Re* of cuttlefish ranges from 100 in newly hatched animals to 20,000 in mature adult animals (Aitken and O'Dor, 2004). Therefore, cuttlefish experience different flow regimes during their development, with hatchling cuttlefish experiencing intermediate flow regimes (*Re*=100–1000), where both viscous and inertial forces are important, and the larger juvenile and adult animals experience flow regimes dominated by inertial forces. Staaf et al. (2014) investigated the effects of mantle length on whole-cycle propulsive efficiency of Humboldt squid (*Dosidicus gigas*) using kinematics and a theoretical model. The model indicated that whole-cycle propulsive efficiency increased with size up to a mantle length of 1 cm, but then declined slightly in larger squid because of the decrease in mantle strain (Staaf et al., 2014; Thompson and Kier, 2001). The scaling of propulsive efficiency may depend on a dimensionless parameter known as the Strouhal number (*St*) that describes the kinematics of the movement (Triantafyllou et al., 1991). There is a relatively narrow range of *St* across which propulsive efficiency is high (0.2<*St*<0.4; Taylor et al., 2003), which is determined by a trade-off between fluid drag and the power required to generate thrust (Floryan et al., 2018; Taylor, 2018). Hence, as *Re* increases, the optimal *St* is expected to decrease to maintain peak propulsive efficiency.

Squid are negatively buoyant and at slow speeds must angle their jet downwards (Anderson and Grosenbaugh, 2005). As speed increases, jet angle decreases owing to the increased lift generated by the body and propulsive efficiency increases. The inverse relationship between propulsive efficiency and jet angle (Anderson and Grosenbaugh, 2005) suggests that neutrally buoyant taxa such as cuttlefish may benefit from a higher efficiency, as they will not have to angle their jets downward to maintain their vertical position in the water column.

This study aimed to build upon the current understanding of cephalopod hydrodynamics by investigating the jet propulsion swimming of the European common cuttlefish (*S. officinalis*). The key aim of this work was to investigate the hydrodynamics of cuttlefish jet propulsion swimming, through the quantification of wake structure and whole-cycle propulsive efficiency in cuttlefish during early ontogeny. We calculated whole-cycle propulsive efficiency as the total mechanical input is expected to be the primary determinant of the overall metabolic cost of swimming. Based upon previous work, it was hypothesised that: (i) the wake structure of cuttlefish jets would fall into discrete categories as described in other jet-propelled organisms; (ii) the jet propulsion swimming of cuttlefish would be more efficient than that of negatively buoyant loliginid squid as a result of their neutral buoyancy; and (iii) because our animals exceed a mantle length of 1 cm, rather than increasing with animal size and *Re*, we predicted that whole-cycle propulsive efficiency would slightly decrease and that *St* would decrease with increased animal size (increased *Re*) as a result of an ontogenetic decrease in the relative amplitude of mantle contraction.

## MATERIALS AND METHODS

### Animals

European common cuttlefish (*Sepia officinalis* Linnaeus 1758) eggs were taken as by-catch upon fishing gear by: (i) JHC research, Poole, Dorset, UK; (ii) The Native Marine Centre, Weymouth, Dorset, UK; (iii) Centre de Recherches en Environnement Côtier, Université de Caen, Luc sur Mer, Normandie, France; and (iv) RK Stride, Christchurch, Dorset, UK, during June 2015 (i, ii and iii) and May 2016 (iv) in the English Channel. Eggs were housed in recirculating artificial saltwater systems at the University of Leeds at a temperature of 19±1°C to maximise development speed while avoiding premature hatching (Bouchaud, 1991). Salinity was maintained at 32±1 PSU using Aqua One Reef synthetic (Kong's, Sydney, NSW, Australia) mixed in deionised water. During egg incubation, additional strontium (6 g per 150 l; Seachem Reef Advantage Strontium, Seachem Laboratories, Madison, GA, USA) was added to tanks to ensure normal statolith and cuttlebone development (Hanlon et al., 1989). Once eggs began hatching, the temperature was gradually (over a period of 10 days) decreased to 15±1°C. Animals were fed twice daily using size-appropriate live foods: live enriched *Artemia salina* (Vitalis live food enrichment, World Feeds Ltd, Thorne, Derbyshire, UK; Peregrine Livefoods, Magdalen Laver, Essex, UK), *Mysis* shrimp (*Mysis spp*.; Aquadip VOF, Oss, North Brabant, The Netherlands; Essex Marine Aquatics, Wickford, Essex, UK) and river shrimp (*Palaemon varians*; Aquatic Live fish foods, Woodford, London, UK). Cuttlefish used in experiments were either hatchlings (<7 days old at the time of experiments) or juveniles (3 months old at the time of experiments).

### Animal housing facilities

Cuttlefish were housed in recirculating, artificial saltwater with a temperature of 15±1°C and salinity of 32±1 PSU (Cefas, 2012) formulated using Aqua One Reef synthetic mixed in deionised water. Animals were housed in size-matched groups in 500, 350 and 300 litre (1300×800×460, 910×690×570 and 890×590×550 mm length×width×height) aquaria, with each tank holding up to 150 hatchling animals, or 50 juvenile animals.

### Wake visualisation and analysis

Individual cuttlefish were captured and transferred to an experimental tank: hatchlings to a 44 litre (460×310×310 mm) tank, and juveniles to a 126 litre (610×460×450 mm) tank. Water temperature and salinity in the experimental tanks matched those of the holding tanks (water temperature of 15±1°C and salinity 32±1 PSU). The water was seeded with aluminium oxide particles (Acros Organics, Pittsburgh, PA, USA) to enable visualisation of the wake (mean particle size 5 µm; seeding density of 30 mg l^−1^; following Dabiri, 2006). Cuttlefish were induced to swim following Karson et al. (2003), where animals were placed inside a tunnel composed of a Perspex^®^ back and base, and black plastic sides (hatchling, 160×55×60 mm; juvenile, 250×100×100 mm), positioned at the top of the tank. Animals were gently encouraged towards the edge of the tunnel, which encouraged animals to spontaneously swim horizontally through the tank (Karson et al., 2003).

**Fig. 1.**
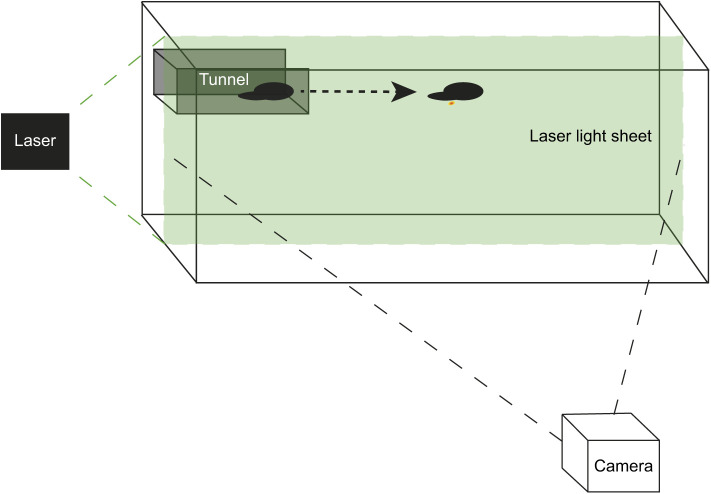
**Schematic diagram showing the PIV system setup.** Cuttlefish were placed in a tunnel, composed of a clear Perspex^®^ base and rear and solid black plastic sides, at the top of the laser light sheet and encouraged to swim out of and through the tank. A camera was placed perpendicular to the tank and used to record at 500 frames s^−1^. A 1 W continuous green laser was used to illuminate aluminium oxide particles suspended in the water.

Visualisation of the jet structure in the sagittal plane of both cuttlefish groups was achieved using a 1 W continuous green (532 nm) laser (Shanghai Dream Lasers Technology Co., Ltd, Shanghai, China) directed through a Powell lens (Thorlabs, Inc., Newton, NJ, USA), creating a 1 mm thick vertical light sheet (following Neil and Askew, 2018). Each cuttlefish and its wake were recorded using a high-speed camera (FASTCAM SA3, Photron USA, San Diego, CA, USA; recording 1024×1024 pixels at 500 frames s^−1^ and shuttered at ^1^/_500_ frames s^−1^), orientated with the recording plane parallel to the laser light-sheet (Fig. 1).

Fluid movements were determined by recording illuminated particles (PIVlab v1.41; Thielicke, 2014; Thielicke and Stamhuis, 2014a,b; MATLAB R2017a, The MathWorks Inc., Natick, MA, USA). Prior to detailed analysis, sequences were pre-processed using a contrast-limited adaptive histogram equalisation tool, enhancing contrast. Next, data were smoothed using the smoothn function (Damian–Garcia smoothing) and adaptive multi-pass processing. This used a total of three passes to track particle movements. This was achieved using nested interrogation windows, where the initial window was 64×64 pixels, the second was 32×32 pixels and the final integration window was 16×16 pixels; this gave a 50% overlap with each interrogation step. A standard deviation filter was used to remove vectors that were more than seven deviations away from the mean flow of the jets. Missing vectors were interpolated using a boundary value solver, giving a smooth interpolation that tended towards the average boundary velocities. As part of the pre-processing steps, the animal was masked using the in-built masking tool in PIVlab.

Jet thrust (*T*) is the force imparted to the environment that propels the animal and equals the rate of change of momentum in the surrounding fluid. Thrust was calculated as (Anderson and DeMont, 2000):
(1)

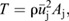
where ρ is the density of seawater (1025 kg m^−3^) and *ū*_j_ is the average jet velocity calculated by taking the time average of the average jet core velocity during the jet period. The core region of the jet was defined as the area of greatest jet velocity. This was determined by running a minimum of four vectors (in the north to south, east to west, northeast to southwest and northwest to southeast directions) through the entirety of each jet to ensure the core velocity was sampled. *A*_j_ is the area of the jet, where measures of jet diameter (*D*_j_) and jet length (*L*_j_) were taken immediately following the release of the jet (following Neil and Askew, 2018), and the area was calculated assuming the jet was cylindrical. Jet area was used as opposed to siphon area owing to the changeable size of the siphon orifice in cephalopods (O'Dor, 1988).

During jet propulsion swimming in cuttlefish, water must be accelerated as it is taken into the mantle cavity and again as it is expelled. The additional kinetic energy that must be given to the water as it is taken into the mantle cavity is not accounted for in the total power requirements of jet propulsion swimming in the calculation of propulsive efficiency (Anderson and DeMont, 2000; Alexander, 2002). For a jet-propelled swimmer with rear intake, the useful power (the rate at which work is being done against drag) is the product of the mass of water propelling the animal per unit time (*m*_j_), animal velocity (*Ū*) and the jet velocity (*ū*_j_), i.e. 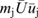; and the total power is calculated as the sum of the useful power (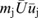), the kinetic energy of the water entering the mantle cavity (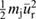) and the kinetic energy given to the water lost in the wake (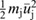) (Alexander, 2002; Neil and Askew, 2018). Therefore, whole-cycle propulsive efficiency can be calculated as (Alexander, 2002; Neil and Askew, 2018):
(2)

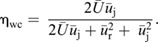


This method is appropriate for cuttlefish because the jetting frequency during swimming is sufficiently high that the fluctuations in swimming velocity can be ignored (i.e. the animal does not come to a standstill in between jets; Alexander, 2002). Refill velocity (*ū*_r_) was estimated following Neil and Askew (2018), assuming the total volume of water ejected is equal to that taken into the mantle during the refill process:
(3)

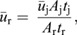


where *t*_j_ is the jet time and *t*_r_ is the refill time. *A*_r_ is the refill orifice area (*A*_r_), which was estimated from the dimensions of the collar flaps (*r*_cf_), assuming the refill orifice area (the collar) is circular:
(4)

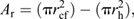


where *r*_h_ is the radius of the head.

Reynolds number was calculated following Alexander (2002):
(5)

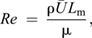
where ρ is the density of seawater at 15°C (1025 kg m^−3^), *L*_m_ is the mantle length and µ is the dynamic viscosity of seawater (0.00115 N s m^−2^).

Strouhal number (*St*; a dimensionless number that describes the cyclical motion of animals) was calculated following Triantafyllou et al. (1991):
(6)

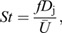
where *D*_j_ is the jet diameter, *f* is the cycle frequency and *Ū* is the animal swimming speed.

Animal swimming speed (*Ū*) was calculated as distance moved (*d*) over the total cycle duration (*t*_cd_=*t*_j_+*t*_r_):
(7)

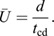


Drag was estimated as:
(8)

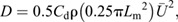
where *C*_d_ is the drag coefficient; here, we used a standard drag coefficient of 0.04, assuming cuttlefish to have a streamlined body shape.

### Statistical analysis

Statistical tests were conducted in R 3.1.1. All swimming sequences were used in data analysis. All data were tested for normality and homogeneity prior to statistical analysis. Where models were used, quantile–quantile (q–q) plots of model residuals were checked to ensure these fit the normal distribution. A critical *P*-value of 0.05 was used to indicate significant differences between models and null models. Parametric tests were used on all data that met the assumptions of normality. Data which did not meet the assumptions of normality were log or arcsine transformed to meet these assumptions. To obtain statistical significance, data were fit to general linear models using R (Bates et al., 2015; Kuznetsova et al., 2017). These models included individual cuttlefish as a random factor, accounting for instances of repeated measures; this ensured all jets produced by each cuttlefish were included in analysis. The statistical significance of these models was determined using analysis of deviance (AOD), where fitted models were compared against null models. Curve fitting was carried out using MATLAB R2021b, with fits optimised to minimise sum of squares errors. The significance of fitted regressions was determined using *t*-tests. Where regression fits were compared, likelihood ratios were used.

## RESULTS

### Animal morphology and swimming kinematics

A total of 244 jet events (from 124 unique sequences) were obtained from 38 hatchling (7.9–12.8 mm; mean±s.d. of 6±4 jets per animal, and 2±2 jets per sequence) and 17 juvenile cuttlefish (20.8–40 mm; 3±2 jets per animal and 2±1 jets per sequence). Animals exhibited two swimming orientations, anterior-first (AF) and posterior-first (PF), in both age groups. PF swimming was associated with greater absolute swimming velocities than AF in hatchling animals (AOD *X*^2^=35.64, d.f.=1, *P*<0.001); there were no differences in swimming velocity between the two orientations in juveniles (AOD *X*^2^=0.04, d.f.=1, *P*=0.84). Hatchlings swam at greater relative speeds than juveniles (AF: ∼150% faster; PF: ∼226% faster) in both orientations (AOD *X*^2^=47.29, d.f.=2, *P*<0.001; see Table 1, Fig. 1). Hatchling cuttlefish had significantly lower *Re* than juveniles (435–787 in hatchlings, 2325–2536 in juveniles; AOD *X*^2^=54.89, d.f.=1, *P*<0.001; Table 1). *St* were significantly higher in hatchlings compared with juveniles (0.38–0.47 in hatchlings, 0.14–0.18 in juveniles; AOD *X*^2^=39.34, d.f. 1, *P*<0.001). Thrust was higher in juveniles (7.29–7.75 mN) compared with hatchlings (0.19–0.30 mN; AOD *X*^2^=23.69, d.f.=1, *P*<0.001), but was unaffected by swimming orientation (AOD *X*^2^=0.09, d.f.=1, *P*=0.77; Table 1).

**Table 1.**
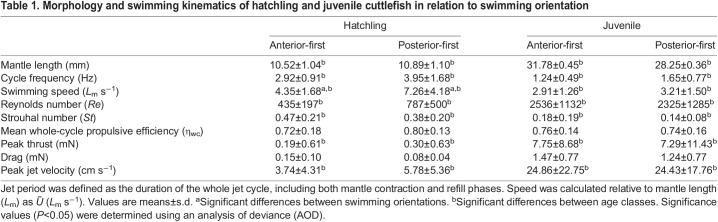
Morphology and swimming kinematics of hatchling and juvenile cuttlefish in relation to swimming orientation

A subsample of sequences obtained from juvenile animals was used (*n*=10 individuals) to obtain more detailed kinematics. The mean duty cycle (the proportion of the swimming cycle made up of the power stroke) was 0.52±0.25 (0.23–0.66), with the mantle contraction period lasting 0.44±0.32 s (0.16–1.13 s; defined as the period over which a jet was being produced) and mantle refill lasting 0.39±0.22 s (0.11–0.86 s; assumed to be the period between jet events). The duty cycle did not differ significantly between swim orientations (AOD *X*^2^=1.92, d.f.=1, *P*=0.166).

**Fig. 2.**
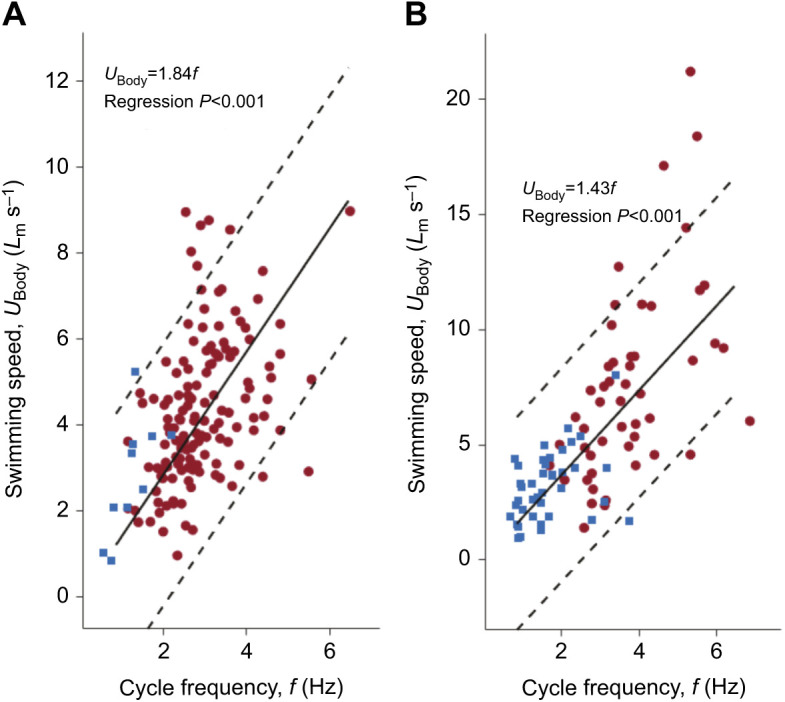
**Relationship between cycle frequency, animal swimming speed and swim orientation.** Regression fits are shown in black with 95% CI shown as dashed lines. Juveniles (*n*=17) are shown in blue, and hatchlings (*n*=38) in red. (A) Posterior-first swimming. (B) Anterior-first swimming. Both fits were significant (*P*<0.001), but fits for anterior- and posterior-first swimming did not differ significantly from one another (*P*=1).

Swimming speed increased with increasing jet cycle frequency (Fig. 2). Regressions were statistically significant (AF: *t*=25.50, d.f.=151, *P*<0.001; PF *t*=18.07, d.f. =91, *P*<0.001); however, no significant differences between AF or PF regressions were noted (*G*=151.39, d.f.=1, *P*=1).

### Wake structure

**Fig. 3.**
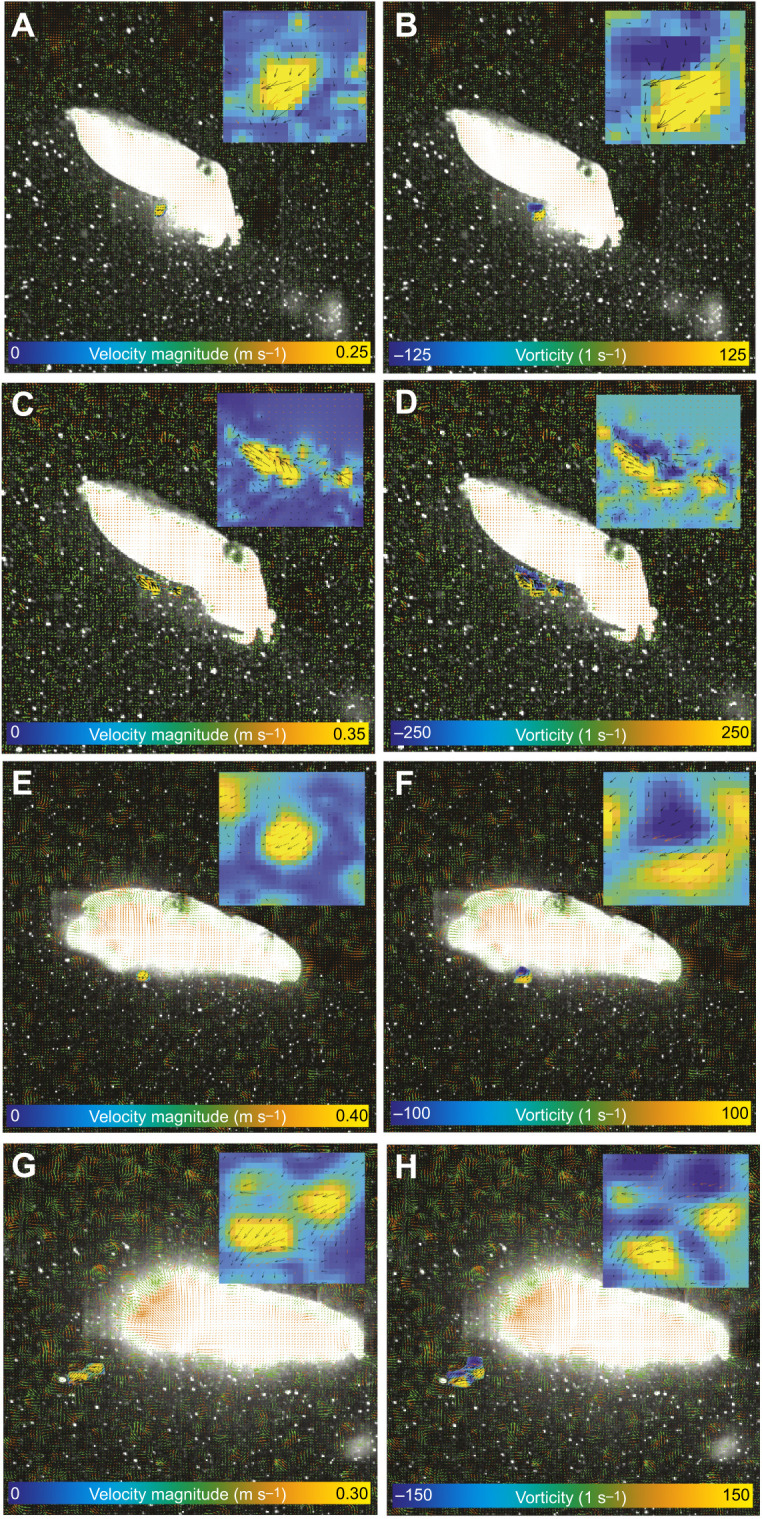
**Comparisons of instantaneous flow and vorticity between jet modes I and II in juvenile cuttlefish.** (A,B,E,F) Jet mode I; (C,D,G,H) jet mode II. (A–D) Animals swimming in the anterior-first orientation, (E–H) animals swimming in the posterior-first orientation; examples used are for illustrative purposes only to enable differences in wake structures to be visualised. Note that the fluid is rolled into an isolated vortex ring during mode I jets (A), whereas the vortex ring is more elongated during mode II jets (B). Blue and yellow regions on vorticity plots denote clockwise and counter clockwise rotation of water, respectively.

**Fig. 4.**
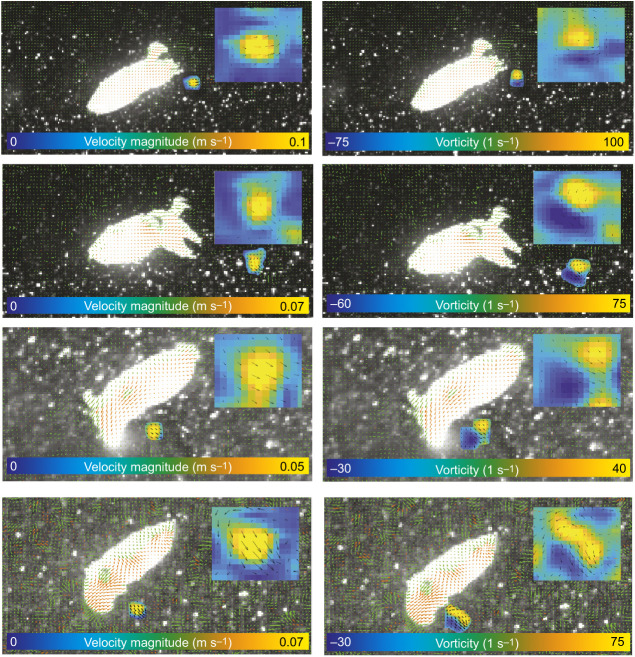
**Comparisons of instantaneous flow and vorticity in hatchling cuttlefish between posterior-first and anterior-first swimming orientations.** Example velocity (A,C,E,G) and vorticity (B,D,F,H) plots from hatchling animals swimming in the posterior- (A–D) and anterior-first (E–H) orientations. Superimposed images shown in the upper left of each panel show the jet structure in further detail.

Two categories of jet were produced: the first consisted of a single isolated vortex, whereas the second consisted of a leading jet vortex followed by a trailing jet (Fig. 3). These two jet structures were termed jet modes I and II following previous nomenclature (Bartol et al., 2008, 2009a,b). These jet modes were seen in both hatchling and juvenile animals, but hatchling animals rarely used jet mode II (5% of jets were categorised as jet mode II; see Fig. 4 for example jet structures). Juveniles utilised both jet modes, with 42% categorised as jet mode I and 58% as jet mode II. Descriptions of, and any comparisons between, jet modes are for juvenile animals only owing to the disparity in jet mode use in hatchling animals. The *L*_j_/*D*_j_ for juvenile animals was higher in jet mode II compared with jet mode I (jet mode I, 3.69±0.28; jet mode II, 5.31±0.28; AOD *X*^2^=8.87, d.f.=1, *P*=0.003). Jet mode did not significantly affect aspects of locomotor performance, with mean peak thrust of 2.96±0.73 and 3.92±1.68 mN (AOD *X*^2^=0.28, d.f.=1, *P*=0.60), and swimming speeds of 2.64±0.30 and 3.28±0.38 BL s^−1^ (AOD *X*^2^=2.33, d.f.=1, *P*=0.13) in modes I and II, respectively.

### Whole-cycle propulsive efficiency

**Fig. 5.**
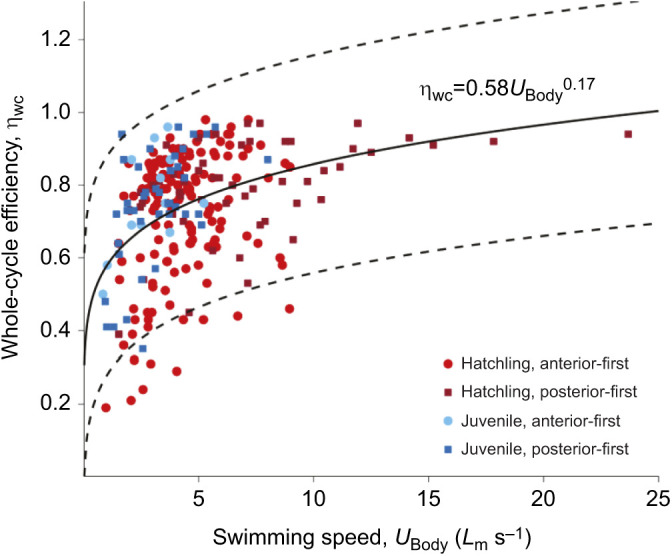
**Relationship between cuttlefish swimming speed and whole-cycle propulsive efficiency in hatchlings (*n*=38) and juveniles (*n*=17).** Relationship is fit in the form of *aU^b^*, and displayed in black, 95% confidence intervals are displayed as dashed lines, *P*-value of fit was 0.005.

Whole-cycle propulsive efficiency was unaffected by swimming orientation, with mean values ranging from 72 to 80% in hatchlings and 74 to 76% in juveniles (Table 1). Jet mode had no significant impact on whole-cycle propulsive efficiency, with mean whole-cycle propulsive efficiency ranging from 69±5 to 72±5% in mode II and mode I jets, respectively (AOD *X*^2^=0.30, d.f.=1, *P*=0.58). Whole-cycle propulsive efficiency in both hatchling and juvenile animals increased with increasing swimming speed as η_wc_=0.58*U*^0.17^ (Fig. 5), regardless of swimming orientation. At greater swimming speeds, such as those exceeding approximately 5*L*_m_ s^−1^, whole-cycle efficiencies generally exceeded 50%, with the majority of animals utilising posterior-first swimming at these speeds. At slower speeds, whole-cycle propulsive efficiency was more variable and tended to be associated with anterior-first swimming (Figs 3 and 4). Despite this relationship, no relationship between cycle frequency and whole-cycle propulsive efficiency was noted.

## DISCUSSION

### Wake structure

Cuttlefish were found to swim using two types of jet structure (Bartol et al., 2009b), where the fluid was ejected either as an isolated vortex ring (mode I) or as elongated jets (mode II). The two jet types observed in cuttlefish have been observed in a variety of species that utilise jet propulsion swimming, such as brief squid (*L. brevis*; Bartol et al., 2009b), chambered nautilus (*N. pompilius*; Neil and Askew, 2018) and king scallops (*P. maximus*; Neil, 2016). Juvenile animals routinely used both types of jet structure, whereas hatchings used predominantly mode I jets. During ontogeny, squid have been shown to use both types of jet structure (Bartol et al., 2009a,b) with mode II jets being employed more frequently than mode I jets, though the proportion of mode I jets was higher in smaller animals, with paralarvae using predominantly mode I jets (Bartol et al., 2009a). The jet mode used during swimming was not related to swimming performance or whole-cycle propulsive efficiency in cuttlefish, as previously demonstrated in nautilus (Neil and Askew, 2018), whereas in squid, mode II jets were associated with higher thrust and mode I jets with a higher propulsive efficiency [although the difference in mean propulsive efficiency was relatively small (10%) and there was considerable overlap in the range between the two jet modes (Bartol et al., 2009b)]. Bartol et al. (2009b) noted that the higher propulsive efficiency observed in animals using mode I jets could have been due to the increased contribution of the fins to propulsion when mode I jets were used. We observed that cuttlefish did not use their fins to propel themselves during the relatively fast (>1.5*L*_m_ s^−1^) jet propulsion swimming, though this has been reported during jet propulsion at slower speeds (below 1.2*L*_m_ s^−1^; Aitken and O'Dor, 2004).

Linden (2011) used a piston to generate jets and demonstrated that the mode of jet produced depends on how much, and how quickly, energy is added to the fluid; each vortex ring can only accept a finite amount of energy, once this is reached, the vortex ring is ‘pinched off’, with the remaining fluid forming an elongate, or trailing, jet. The physical limit to the size of vortex ring that can be formed has been demonstrated to occur when the ratio of the length of the jet to its diameter (*L*_j_/*D*_j_) is ∼4 (Gharib et al., 1998; Bartol et al., 2009b); above this relative size, a trailing jet (mode II) is produced. In juvenile animals, the *L*_j_/*D*_j_ of mode I jets was 3.69 and that of mode II jets was 5.31, which is similar to those of other jet propulsion swimmers (e.g. nautilus and squid, where the transition from mode I to mode II jets occurred at *L*_j_/*D*_j_∼3; Neil and Askew, 2018; Bartol et al., 2009b) and consistent with limiting *L*_j_/*D*_j_ in mode I jets of found in mechanically generated jets (*L*_j_/*D*_j_∼4; Gharib et al., 1998).

Trailing jet vortices exhibit increased turbulence, which reduces the energy within the jet that can be transferred into useful momentum, and hence reduces the whole-cycle propulsive efficiency (Dabiri et al., 2010; Linden and Turner, 2004). The jet mode used during swimming was not related to swimming performance or whole-cycle propulsive efficiency in cuttlefish, as previously demonstrated in nautilus (Neil and Askew, 2018), whereas in squid, mode II jets were associated with higher thrust and mode I jets with a higher whole-cycle propulsive efficiency, although the difference in mean efficiency was relatively small (10%) and there was considerable overlap in the range between the two jet modes (Bartol et al., 2009b). Bartol et al. (2009b) noted that the higher propulsive efficiency observed in animals using mode I jets could have been due to the increased contribution of the fins to propulsion when mode I jets were used.

The structure of the jets is also related to animal size (Anderson and Grosenbaugh, 2005; Herschlag and Miller, 2011): newly emerged cuttlefish used jet mode I more than 90% of the time, whereas juvenile animals routinely used both types of jet structure. Similar patterns in jet mode utilisation have been noted during ontogeny in brief squid (*L. brevis*) and longfin squid (*D. pealeii*), where hatchlings predominantly utilised jet mode I, with increased reliance upon jet mode II with increasing size (Bartol et al., 2009a,b; Anderson and Grosenbaugh, 2005). These studies suggest that animals transition from using jet mode I to using jet mode II with increased size.

### Whole-cycle propulsive efficiency

The average whole-cycle propulsive efficiency of jet propulsion swimming in both hatchling and juvenile cuttlefish across all speeds was 72–80%. The average whole-cycle propulsive efficiency in cuttlefish was similar to the upper end of the range measured in nautilus (30–76%; Neil and Askew, 2018) but higher than has been previously reported in squid (38–49%; Anderson and Grosenbaugh, 2005 recalculated from Anderson and DeMont, 2000), salps (47–55%; Sutherland and Madin, 2010) and jellyfish (53%; Neil and Askew, 2018), calculated using a similar approach (i.e. accounting for the energy losses associated with the uptake of fluid into the mantle cavity). Other studies in which efficiency has been calculated based on time-averaged thrust and excess kinetic energy in the wake have also shown squid can achieve relatively high propulsive efficiencies (>80%; Anderson and Grosenbaugh, 2005; Bartol et al., 2009a,b, 2016). Therefore, despite the perceived inefficiencies of swimming by jet propulsion (Krieg and Mohseni, 2015; O'Dor and Webber, 1991; Weymouth and Triantafyllou, 2013), it appears that in some instances the whole-cycle propulsive efficiency of cephalopod molluscs can be relatively high. This could be related to whether they are negatively buoyant (e.g. loliginid squids) or neutrally buoyant (e.g. cuttlefish and nautilus), because jets must be generated at an angle in order to support body weight in negatively buoyant species at slow speeds, and propulsive efficiency decreases with increasing jet angle (Anderson and Grosenbaugh, 2005).

One key component of whole-cycle methodologies is the refill velocity. Unlike jet velocities, estimating refill is more difficult in freely swimming animals, as refill cannot be easily visualised. Here we estimated refill through measurements of the collar and head. This was then used to calculate the refill area assuming a circular cross-section. Anderson and Grosenbaugh (2005) approached this in a different way, instead assuming the refill area would be approximately 2–3× the jet area. Both approaches introduce potential error; however, our approach is less arbitrary and assumes the refill area is linked to the area of the collar flaps. We also note both Anderson and Grosenbaugh (2005) and Anderson and DeMont (2000) calculated the whole-cycle frequency using a different approach to the one employed here. Anderson et al.'s approach (Anderson and Grosenbaugh, 2005; Anderson and DeMont, 2000) introduces a theoretical upper limit of 58% and may in part explain the lower efficiencies reported when employing this methodology. A comparison of our results calculated using these two methodologies is shown in Fig. S1 and reveals that estimates using Anderson et al.’s approach (Anderson and Grosenbaugh, 2005; Anderson and DeMont, 2000) are substantially lower. The approach employed here does not place an upper limit on how efficiently an animal can swim, instead looking solely at the ratio of useful power output to total power input. While both approaches have their merits, the limits placed using Anderson et al.’s approach may lead to underestimating the efficiency.

Whole-cycle propulsive efficiency increased concomitantly with swimming speed, from 58% at 1 BL s^−1^ to 86% at speeds of 10 BL s^−1^ (estimated using equation in Fig. 5). A similar relationship between efficiency and speed has also been reported in squid (Anderson and Grosenbaugh, 2005), and nautilus during posterior-first swimming (49% at 0.5 BL s^−1^ to 62% at 1.5 BL s^−1^; calculated using the equation in fig. 5C of Neil and Askew, 2018). Efficiency increased with swimming speed during posterior-first swimming in cuttlefish, nautilus and squid (*L. brevis*; Bartol et al., 2008, 2009b); efficiency also increased with speed in anterior-first swimming in cuttlefish and squid but not in nautilus, where whole-cycle propulsive efficiency decreased with increasing anterior swimming speed (Neil and Askew, 2018). In hatchling cuttlefish, swimming speed was higher during posterior-first swimming than during anterior-first swimming. The siphon is bent back on itself during anterior-first swimming; this could result in turbulence in the fluid flowing through it, which may decrease the useful energy transferred into the jet (Keulegan and Beij, 1937; Vogel, 1994), though unlike in nautilus during anterior-first swimming, whole-cycle propulsive efficiency was unaffected (Neil and Askew, 2018).

Whole-cycle propulsive efficiency did not vary between the two jet modes as found previously in jet propulsion swimming in nautilus when swimming with a particular orientation (Neil and Askew, 2018). However, in squid and mechanically generated jets, mode II jet structures have been found to be associated with increased drag, which reduces both propulsive efficiency and thrust (Bartol et al., 2009b; Linden, 2011), though the increase in drag in squid could be due to undulatory fin movements affecting flow (Bartol et al., 2009b; note that undulatory fin movements were not observed during cuttlefish jet propulsion swimming). The lack of differentiation between jet modes in cuttlefish and nautilus suggests that animals can compensate for the theoretical inefficiencies of mode II jets in some way.

The differences observed in both the structure of jets, as well as swimming speeds, thrust and ultimately whole-cycle propulsive efficiency, are likely also influenced by the hydrodynamic forces experienced by animals. It was hypothesised that both whole-cycle propulsive efficiency and *St* would decrease slightly with increasing *Re* (i.e. increasing body size) based on the predictions of Staaf et al. (2014). Hatchlings had an *Re* ranging between 435 and 787, and juveniles had an *Re* of between 2325 and 2536. However, there was no significant difference in whole-cycle propulsive efficiency in hatchlings compared with juveniles. Although a slight decrease in whole-cycle propulsive efficiency was hypothesised (based on a theoretical model of jet propulsion swimming in squid; Staaf et al., 2014), in squid the predicted decrease in whole-cycle propulsive efficiency across a similar size range to our cuttlefish is relatively small, ∼7% of the peak efficiency, which may be why we were unable to detect this empirically. The increase in *Re* during ontogeny represents the increase in the relative importance of the inertial forces. Floryan et al. (2018) suggested that below an *Re* of 1000, the *St* at which maximal propulsive efficiency occurs decreases with decreasing drag. Therefore, as the relative importance of drag depends on *Re*, it is expected that in smaller animals the observed *St* will decrease with increasing size (for *Re*<1000). The fall in *St* with increased *Re* during ontogeny in cuttlefish supports our hypothesis (Floryan et al., 2018): *St* ranged from 0.38–0.47 in hatchlings to 0.14–0.18 in juveniles. The decrease in *St* with increasing *Re* has been observed in rainbow trout (Webb et al., 1984), and as a general trend across species (Kayan et al., 1978). These data suggest that the morphology of the cuttlefish jet propulsion system and body have been tuned by natural selection such that the *St* at which thrust and drag are balanced is that at which whole-cycle propulsive efficiency is maximal (Taylor, 2018).

### Conclusions

The volume of fluid that can be ejected to provide thrust by a jet-propelled swimmer is limited by the volume of the animal, which makes this mode of swimming relatively energetically expensive. A comparison between the metabolic cost of jet propulsion and undulatory swimming (Bartol et al., 2001; Krieg and Mohseni, 2015; O'Dor and Webber, 1991; Weymouth and Triantafyllou, 2013) supports this notion, with fish having a lower metabolic cost of transport than a similarly sized squid. However, this study on cuttlefish and previous work on nautilus (Neil and Askew, 2018) and squid (Anderson and Grosenbaugh, 2005; Bartol et al., 2009a,b) indicate that both the whole-cycle and propulsive efficiency in some cephalopod swimmers are relatively high (in excess of 85%), and higher than that estimated in some undulatory swimmers (propulsive efficiencies of ∼45%; Maertens et al., 2015). The whole-cycle propulsive efficiency is only one component in the transduction of chemical energy into useful energy in the environment – other components include the efficiency of ATP synthesis from substrates and ATP utilisation by the locomotory muscles; therefore, it is possible that the relatively high metabolic cost of transport arises from one of these steps being relatively inefficient. Also, the previous hydrodynamic arguments as to why undulatory swimmers are more efficient than jet-propelled swimmers have not considered the drag amplification owing to body undulations that reduces the efficiency of undulatory swimmers (Maertens et al., 2015).

## Supplementary Material

10.1242/jexbio.246225_sup1 Supplementary information
