# Supplementary material for: The hydrodynamics of jet propulsion swimming in hatchling and juvenile European common cuttlefish, Sepia officinalis
Source: J Exp Biol. 2023 Sep 28;226(18):jeb246225. doi: 10.1242/jeb.246225 (PMC10560557; doi:10.1242/jeb.246225)
Supplement: Supplementary information [file jexbio-226-246225-s1.pdf]

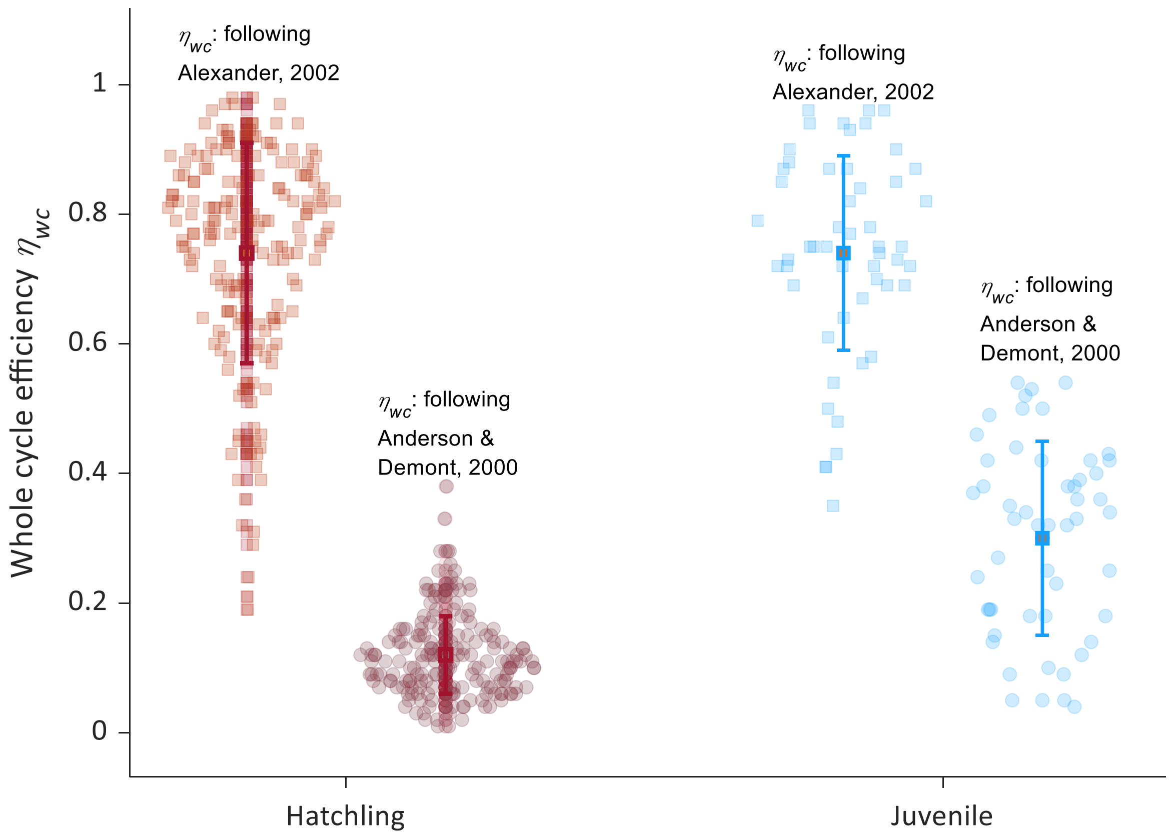

Fig. S1. Comparison between the results obtained from the two whole-cycle efficiency methodologies. The methodology employed by Alexander (2002) returned greater efficiencies than the approach employed by Anderson and DeMont (2000).
